# Towards a secure cloud repository architecture for the continuous monitoring of patients with mental disorders

**DOI:** 10.3389/fdgth.2025.1567702

**Published:** 2025-06-30

**Authors:** Dimitra Georgiou, Stamatis Katsaounis, Panayiotis Tsanakas, Ilias Maglogiannis, Parisis Gallos

**Affiliations:** ^1^ Department of Digital Systems, School of Information and Communication Technologies, University of Piraeus, Piraeus, Greece; ^2^ Department of Electrical & Computer Engineering, School of Electrical and Computer Engineering, National Technical University of Athens, Athens, Greece

**Keywords:** healthcare platforms, security, cloud computing, elasticsearch, sensors

## Abstract

**Introduction:**

Advances in Information Technology are transforming healthcare systems, with a focus on improving accessibility, efficiency, resilience, and service quality. Wearable devices such as smartwatches and mental health trackers enable continuous biometric data collection, offering significant potential to enhance chronic disorder treatment and overall healthcare quality. However, these technologies introduce critical security and privacy risks, as they handle sensitive patient data.

**Methods:**

To address these challenges, this paper proposes a security-by-design cloud-based architecture that leverages wearable body sensors for continuous patient monitoring and mental disorder prediction. The system integrates an Elasticsearch-powered backend to manage biometric data securely. A dedicated framework was developed to ensure confidentiality, integrity, and availability (CIA) of patient data through secure communication protocols and privacy-preserving mechanisms.

**Results:**

The proposed architecture successfully enables secure real-time biometric monitoring and data processing from wearable devices. The system is designed to operate 24/7, ensuring robust performance in continuously tracking both mental and physiological health indicators. The inclusion of Elasticsearch provides scalable and efficient data indexing and retrieval, supporting timely healthcare decisions.

**Discussion:**

This work addresses key security and privacy challenges inherent in continuous biometric data collection. By incorporating a security-by-design approach, the proposed framework enhances trustworthiness in healthcare monitoring technologies. The solution demonstrates the feasibility of balancing real-time health monitoring needs with stringent data protection requirements.

## 1 Introduction

**Figure 1:**
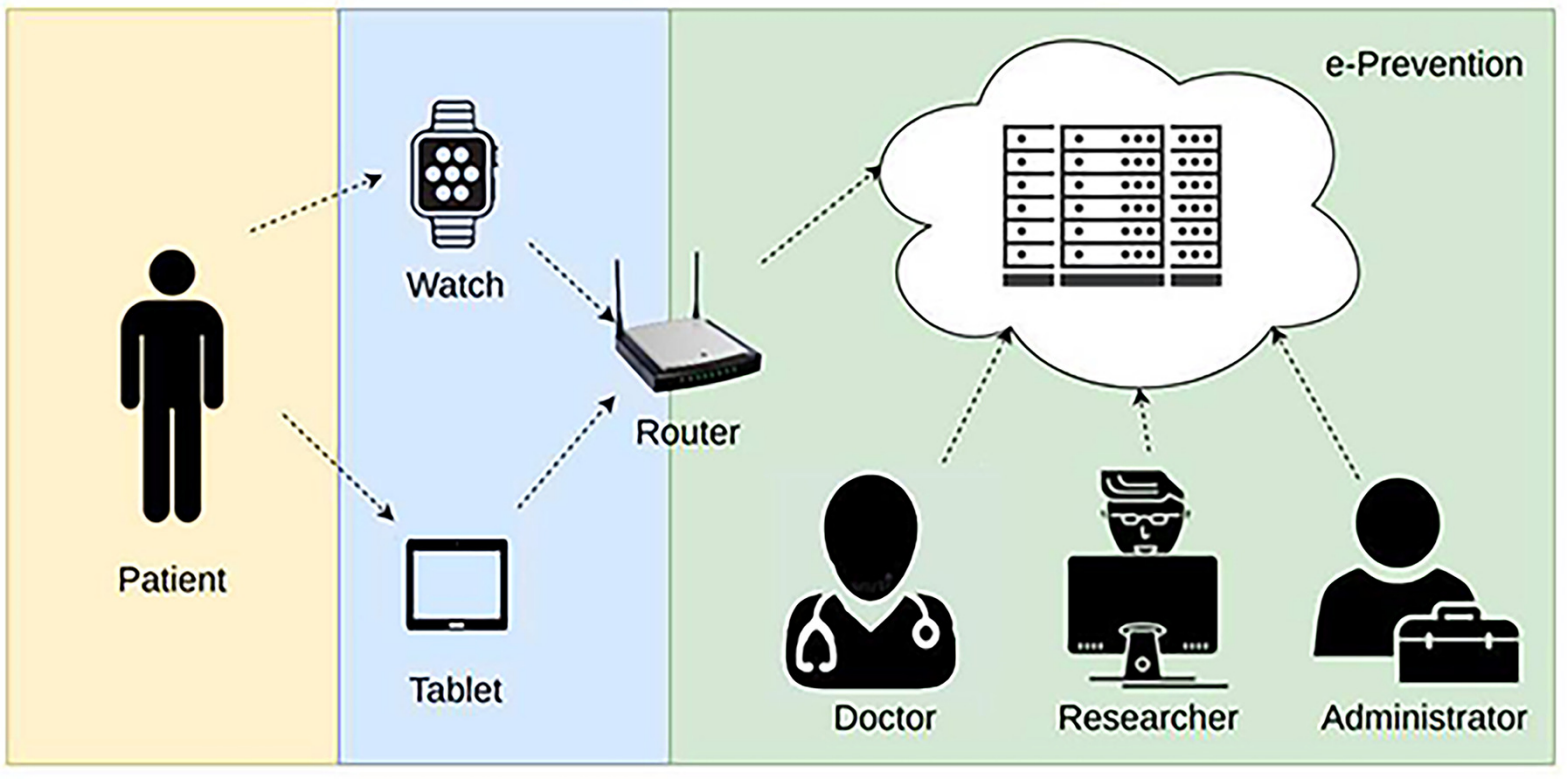
A typical remote health monitoring system.

The Wireless Body Area Networks (WBAN) are considered advanced key technologies to provide protection from chronic diseases, such as mental disorders which, unfortunately, are affecting a lot of people around the globe. The latest World Health Organization (WHO) World Mental Health Report finds that a staggering one billion people, more than one in eight adults and adolescents) worldwide have a mental disorder (1). Mental health has worsened worldwide since the pandemic. In Europe, according to a recent Eurobarometer survey on mental health, conducted in June 2023 revealed that almost 1 in 2 people 46% of Europeans experienced an emotional or psychosocial problem, such as feeling depressed or anxious, in the past twelve months (2). The most prominent of them are psychotic disorders (schizophrenia, bipolar disorder, and others) due to the severity of the symptoms, the high impact they have on patient's functionality and the existing therapeutic approaches. These challenges necessitate improvements in services and new initiatives to enhance mental health care provision. A significant part of these patients show resistance to the prescribed treatment. As mental disorders can affect the quality of life and undermine motivation, an early detection of such diseases can be helpful to provide advance treatment, and thus, minimizing potential complications. For this reason, several researchers are working on deploying an innovative application and advanced computer-based platform, that facilitates the effective monitoring and can support the relapse prevention in such patients. Nowadays, the need of remote health monitoring systems has been increased, where e-health care professionals use portable and wearable sensors, not only to acquire and monitor patients'health status in real time, but also to identify abnormalities in the covered data sets. A typical WBAN-based electronic health monitoring system consists of wearable or implanted sensors, wireless communication infrastructure gateways and remote servers. The WBAN is a set of small and tiny sensors attached to a smartwatch or implanted on a human body to measure health information such as heart rate, temperature, sleep activity, R-R interval, feeling/mood state, perceived pain etc. It sends data via wireless communication infrastructure to the gateway, which serves as a relay node to the monitoring system and then the remote/cloud servers store, process and access the collected data. This data can be accessed by the authorized doctors or any other revenant user, as depicted in Figure 1.

**Figure 2:**
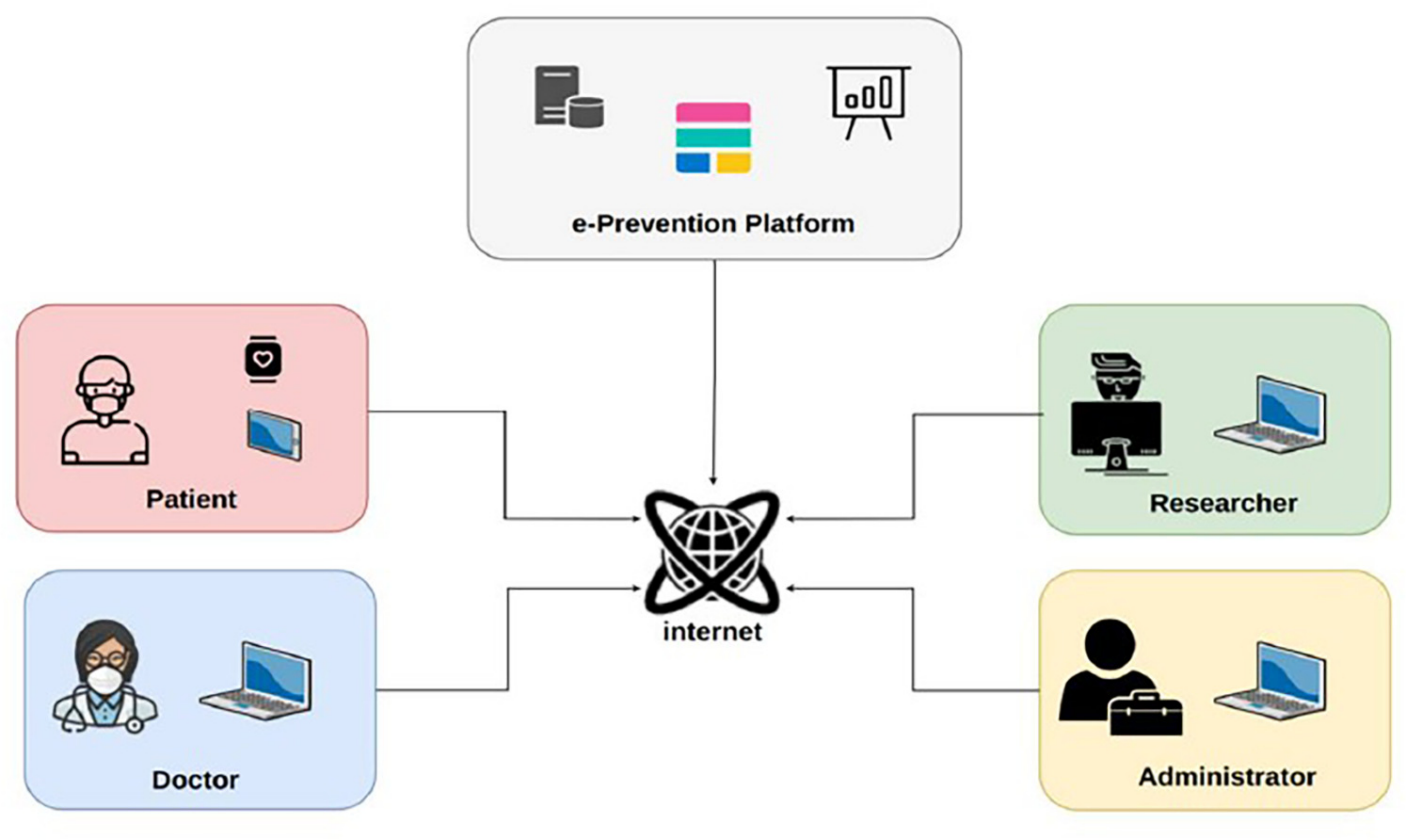
The e-Prevention solution - general idea.

Although real-time monitoring based on portable and wearable sensors can instantly convey patient health status to remote servers for further processing, it also can expose it to malicious intruders. As a result, security is a major concern, as it involves several wireless devices, distributed systems, and non-stationary patients. In this paper, the following research contributions are addressed:

• Analysis of the security—privacy requirements and threats of modern WBANs, paying particular attention to health-related applications.
• Outline of the e-Prevention system in Figure 2 for continuous monitoring, early diagnosis, and treatment of patients with psychotic disorders.
• Presentation of a novel framework for the security and privacy preserving of wireless body sensor network (WBSN) applications in the context of Elasticsearch, along with an outline of the current state-of-the-art methods from literature.

Unlike most existing e-health platforms, which are designed for episodic data collection or short-term monitoring, the proposed system is specifically built to support continuous, 24/7 recording of sensitive biometric and behavioral data using wearable devices such as smartwatches and mental health trackers (3). This constant data flow introduces unique security and privacy challenges that are not adequately addressed by conventional frameworks. The proposed approach stands out by adopting a security-by-design methodology, which incorporates threat modeling, stakeholder analysis, and security requirement specification early in the system's lifecycle. It ensures comprehensive security guarantees, including confidentiality, integrity, availability, non-repudiation, accountability, and data freshness critical for systems operating in real-time collection. Implementation is achieved through a robust Elasticsearch-based infrastructure, enhanced with encrypted communication channels (HTTPS), strict role-based access control (RBAC), continuous data signing and validation, and private internal networking for component communication. By embedding these mechanisms directly into the system architecture, this framework addresses the limitations of prior solutions and provides a resilient foundation for secure, always-on mental health monitoring.

This paper is organized into 7 sections. Section 2 presents a literature review of security and privacy in health systems. Section 3 presents the security issues in WBANs. Section 4 presents the case study of a WBSN healthcare project, the e-Prevention. It discusses the general architecture and functionalities of different sub-components of this system and its requirements, the types of data and the respective data flows. Section 5 presents a comprehensive security and privacy analysis of the Proposed Secure framework. Section 6 presents the features implemented at the e-Prevention system following the proposed framework. Finally, Section 7 discusses the conclusions and future steps.

## 2 Literature review

Following the revolutionary progress in the field of Cloud Computing (4), traditional healthcare systems are being transformed into e-health platforms hosted in Cloud Computing. Despite the obvious benefits of cost-effective storage, flexibility, scalability and ease of data access, e-health platforms face new security and privacy challenges. Health data, usually organized in electronic health records (EHRs), contains sensitive patient information, and as such, are subject to privacy and security mechanisms (5). According to Benson (6), to assure privacy on data of e-health platforms the following minimum requirements should be met (a) user authentication, (b) user authorization, and (c) patient's consent of their health data access by others. One way to ensure that all the above requirements are implemented in a secure way is through cryptographic schemes. In this type of proposed security mechanisms one or more cryptographic methods are deployed to encrypt the data either at rest or in transit as well as a combination of them. In their study, Soceanu et al. (7) proposed a solution, based on the policy platform SAFAX and the cryptographic suite ARCANA. The proposed platform assumes that patients encrypt their health data with their private key and provide access to them to well-specified system entities such as their doctors. The SAFAX platform can ensure that only the appropriate entities can access and decrypt patients' data and can dynamically re-encrypt the data as the access list is altered. The entire process relies on hierarchical key trees. Another e-health platform, strongly relying on cryptography, has been proposed by Dhivya et al. (8). In their study, they introduce a two-level user authentication scheme, based on a combination of biometrics and location, backed up by the Kerberos protocol. In addition, a steganographic technique accompanies the storage layer which guarantees that access is granted only to authorized users. Anonymity is an alternate method of ensuring an e-health platform's security. Since anonymized data can still be read by humans as opposed to being encrypted, some information may be compromised in the event of a successful attack. However, anonymization eliminates the need for much more processing time and power because the data cannot be connected to their real owners. In their study, Galletta et al. (9) propose a secure and efficient way to share magnetic resonance imaging (MRI) data between doctors in the Cloud. The proposed platform consists of two main components, one for anonymizing the data and the other for obfuscating and storing the data in multiple cloud storage providers. Anonymization is achieved by replacing the patient's full name and date of birth with random fields in such a way that doctors can conduct their diagnosis without problem. Obfuscation is relying on data deduplication, a widely used data compression technique. A technique which is widely used as a security measure for data protection is deception technology. In their study, Al Hamid et al. (10) introduced “decoy files” which act as honeypots for malicious attackers. With the use of decoys, storage and sharing of multi-media medical data can be achieved in a secure way in a challenging fog computing paradigm. The decoy technique relies on encryption and the spread of real and decoyed data at the edge of the network. While legitimate users can decrypt the original data with their private keys, an attacker is exposed to decoyed data or data that seem to be real but contain falsified information. In addition, the e-health platform can detect intruders by special headers placed in decoyed files and raise alerts when they attempt to access them. While there are many techniques and methodologies which can boost the security in cloud-based e-health platforms, it is difficult and complex to implement guarantee the absolute level of confidentiality, integrity, and availability of their data. As is highlighted in the work of Altamimi et al. (11), e-health systems are vulnerable to a wide range of security threats because of their portability and distributed design. Attacks can potentially happen in every stage of the data life cycle; at data collection stage, at transmission, and at the storage space where data is kept. To mitigate as many threats as possible, the Context-aware Access Control Security Model (CARE) is introduced. The CARE model acts as a middleware between data and users. Through a role-based access control mechanism, a user can access and share e-health data, with every action being audited. All in all, security in the Cloud, especially when the safety of sensitive data is at stake, is a very crucial process requiring careful design and implementation. This study is going to use a combination of security models and expand to the infrastructure which hosts an e-health Cloud platform and how it can be hardened to increase the overall security of the system. In this regard, E-prevention is, a pioneering system, funded by the European Regional Development Fund of the European Union and Greek national funds through the Operational Program Competitiveness, Entrepreneurship and Innovation, under the call RESEARCH—CREATE—INNOVATE (project code:T1EDK-02890) going beyond previous research, introduces medical wearables to the health mental systems in order to form a strong paradigm of how wearable technology can respect the user's security and privacy. Its mission has been manifolding, aiming at the development of (a) a holistic, unobtrusive, (b) autonomous and (c) security preserving platform for real-time monitoring of patients' daily mental and physiological status. To that aim, it provides a Secure Framework, which suggests the appropriate steps so as technical, organizational, and procedural measures for the satisfaction of the requirements. E-Prevention, through this Framework, covers the gaps in existing methodologies, focusing on the increase of the patients' trustworthiness to the developed software.

## 3 Security and privacy issues

### 3.1 Security and privacy requirements of wireless body sensor network

IT security in healthcare systems, services and applications is a major concern due to the high privacy and confidentiality requirements. In a health application, a Body Sensor Network can be used to collect critical patient health information. This information can be used to drive near real-time monitoring of a patient having various diseases such as asthma, physical disabilities, as well as people with neurological disorders. Considering that health information is a particularly sensitive subset of personal information, security of patient information as well as the privacy are the primary features for any Wireless Body Sensor Network system. Security indicates that patient information is secured from unauthorized access while being exchanged, gathered, processed, and stored securely. Privacy is defined as the process that authoritatively controls and monitors the usage of patient data. Health Sensor Systems must maintain particular security methods to assure confidentiality, integrity, and availability of patient medical information. But, except for the triad “confidentiality, integrity, and availability” (CIA), there are some other security requirements that should be considered. To ensure the safety of a Body Sensor system and its extensive acceptance by its users, we conducted a survey as part of this research, and we identified the following requirements that have already been also addressed by different authors.

• Data Confidentiality: Confidentiality of data is about protecting data against unintentional, unlawful, or unauthorized access, disclosure, or theft. Confidentiality is often equated with privacy protection (12–14). However, although the protection of privacy presupposes confidentiality, it is not an identical concept as the protection of privacy requires the fulfilment of other security requirements. It is considered as the primary issue in the Sensor Network. Health Data needs to be protected from unauthorized access and leaking (15–25).
• Data Integrity: It is associated with the accuracy and consistency (validity) of data over its lifecycle. In health information systems, integrity is applied not only to the transmitted information but also to the contents of the patient's electronic file, either during transmission or during storage. Procedures that ensure integrity protect the user from messages that have been changed, or from the intervention of a malicious user/attacker, or from alterations in communication. As a security requirement, integrity does not directly protect privacy, but it is necessary for the safe operation of a health information system where patient health information should not be compromised under any circumstances. As WBAN applications contain sensitive Personal data, a patient's life could be in danger without the protection of data integrity (12–14, 26).
• Data Availability: refers to the capability to ensure that the data one system needs to function is always accessible when and, where required, even when disruption occurs. Since such a system carries important, extremely sensitive, and potentially lifesaving information, it is required that the network is available at any time (27–29).
• Data Authentication: In health information systems, authentication is an essential security requirement as incomplete authentication mechanisms may allow unauthorized users access sensitive data managed by these systems resulting in privacy threats. In WBAN application, the authentication process will check the identity of a user before allowing access to any Personal data (18, 30–32).
• Authorization: In Body Sensor systems, data authentication is necessary to validate the nodes in use.
• Access control: It is the instrument to control data protection—both in terms of integrity and privacy—and ensure that the user who has access to a specific information is well-trained and able to use it efficiently for the appropriate purpose (24, 33). In a WBS network data can be accessible by multiple entities such as doctors, medical staff, and patients.
• Non-repudiation: It refers to the ability to ensure that the application must accept the authenticity of their signature on a document (cannot deny the authenticity) of a message which originated from it. For this reason, it is necessary to implement mechanisms ensuring that nobody can deny his actions.
• Data Freshness: It ensures that old data is not recycled and that its frames are correct. Data freshness also makes certain that the integrity and confidentiality of data are protected from recording and replaying outdated versions. There are two types of data freshness: a) Strong freshness which guarantees the order of both the frames and the delay b) Weak freshness which only guarantees the order of the frames (24).
• Accountability: It entails the procedures and processes by which one party justifies and takes responsibility for its activities. In WBS networks when a user of the system abuses his privilege and carries out unauthorized actions on patient-related data, he should be identified and held accountable.
• Anonymity: The anonymization of the data prevents the identification of the person to whom the data refers. For a WBAN application Anonymization refers to the processing of personal data in a manner that makes it impossible to identify individuals from them. It protects users' privacy, as a particular individual cannot be linked with the data being stored or transmitted (18, 34).
• Key management: key management is constrained by sensors, computational power, battery capacity, memory, and the transmission range. Generating unique cryptographic keys is the most important challenge to ensure data security (19, 20, 35, 36).

### 3.2 Security and privacy related threats/attacks in WBSNs

Although WBANs improve the quality of patient care, the connected devices use wireless communication protocols to transmit the acquired data. WBANs are susceptible to a wide range of potential problems. In this section, we discuss threats or attacks related to WBANs that would be harmful for the respective healthcare applications. According to the ENISA Glossary, a threat is “any circumstance or event with the potential to adversely impact an asset through unauthorized access, destruction, disclosure, modification of data, and/or denial of service” (37). Attack is a deliberate unauthorized action on a system or asset and can be classified as active or passive (38, 39). An attack will have a motive and will follow a method when opportunity arises. The main difference between threat and attack is that a threat can be either intentional or unintentional whereas an attack is intentional. The vulnerabilities could be weaknesses in the technology, configuration, or security policy. Any discovered vulnerability must be addressed to mitigate any threat that could take advantage of this vulnerability. WBANs are usually more vulnerable to various security threats since unguided transmission is more susceptible to security attacks. A list of the most common threats and attacks on WBANs reported in the literature is presented in this subsection:

• Eavesdropping/Interception: This is the prevalent threat to patient privacy. The attack takes advantage of unsecured network communications to access data as it is being exchanged. Conventional WSNs consist of wireless nodes equipped with antennas, which broadcast radio signals in all directions and are consequently prone to eavesdropping attacks. An attacker can use this data to introduce themselves as an authorized member to launch an impersonation attack (21, 40, 41).
• Spoofing and Sybil attack: When a malicious node masquerades as a legitimate entity of the system to disrupt the network while avoiding detection. Thus, Spoofing refers to the act of posing as someone else (i.e., spoofing a user) or claiming a false identity (i.e., spoofing a process). This category of threat is concerned with authenticity.
• Man-in-the-middle: It requires the attacker to intercept communications between two parties. After inserting themselves in the “middle” of the transfer, the attackers pretend to be both legitimate participants. The attacker can eavesdrop and manipulate the message in real-time without the sender or receiver noticing (30, 42).
• Data/Message Modification: Is an escalation of the eavesdropping attack where the adversary deletes or modifies the information transmitted. This is particularly dangerous in health care where data used is vital to the users' wellbeing (35, 43, 44).
• Masquerade Threat: the attacker aims to masquerade himself/herself as an authorized user to gain higher privileges to access sensitive data. In Healthcare, the attack may be attempted from within the hospital, by a medical faculty member or may originate from an outside client *via* some connection open to the hospital's local network (27, 30, 42).
• Replay Threats: If a masquerade relay node captures the patient physiological data, later, these captured messages can pose replay threats to the real-time healthcare application. As the patient treatment depends on fresh received messages from medical sensor networks, this could cause mistreatment of the patients (15, 18, 19).
• Repudiation: This threat occurs when the receiver denies that an action or an event has occurred, e.g., denies the fact of having received a message in WBANs. In a healthcare WBANs the authorized user performs illegal operations, and the system cannot trace it, and other parties/authorities cannot prove this. This category of threat is concerned with non-repudiation.
• Denial of Services threats/attack: A DoS attack can target the unavailability of the web applications and their associated services in the network (11, 12, 14, 15, 17, 18). In healthcare applications Denial-of-service threat could be even more disruptive, because such a network needs to be always-on (i.e., in-home, in-hospital, etc.). To disable the operation of the sensor, the intruder can just destroy the nodes. Although sensor networks can be restored in case of node failure, the attacker can corrupt the network, destroying many critical nodes.
• Impersonation attack: Allows an eavesdropping attacker to pose as a trusted person authorized member of a network. The attacker obtains private identity information and uses it to impersonate a legitimate node in the network. These attacks are highly targeted and well-crafted to appear realistic and authentic. In healthcare systems such an attack can ask you to take some action to gain access to sensitive information.
• Insider Attack: In WBANs applications insider attackers can use a physically compromised node with authorized system access, to drop, modify and misroute data packets to harm normal network functionality (14, 15, 17). They also may have knowledge of the network setup and vulnerabilities, or the ability to obtain that knowledge, better than anyone on the outside. The Active type of the insider threat is associated with someone internally doing something deliberate that causes harm, while the Passive insider threat deals with users that are ill-informed or with poor security posture.

## 4 The case study of the e-prevention platform

**Figure 3:**
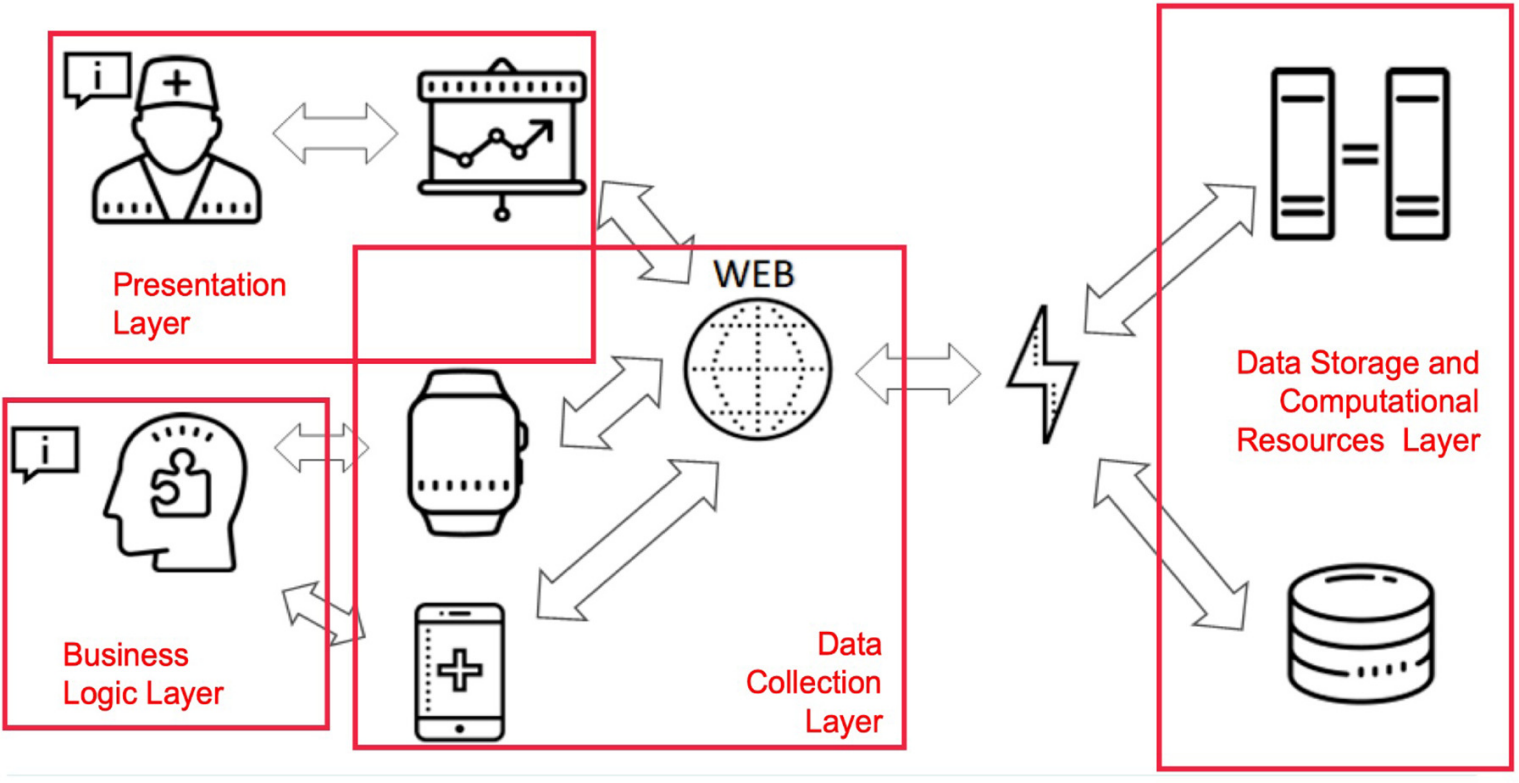
Layered architecture of the e-Prevention platform.

The e-Prevention platform is designed to provide remote care services, adaptable to each participating patient (45). It uses continuous patient monitoring technologies and semi-automatic decision support techniques for the early diagnosis and treatment of patients suffering from psychotic disorders. Figure 3 illustrates the layered architecture of the e-Prevention platform, highlighting the interaction among the presentation, business logic, data collection, and data storage/computation layers.

It is based on off-the-shelf smartwatches with embedded sensors that measure relevant activity signals on a periodic basis. Tablet devices are used for video recordings of patients' meetings with their doctors. At the server side, e-Prevention consists of the following Cloud components: A BioSignal Collection Server which is responsible for collecting smartwatch data. A cold storage server for long-term storage of compressed data. A dashboard server for collecting tablet video data and health metric questionnaires filled with by doctors.

An Elastic Stack (46) installation for data aggregation, visualization, and analysis, consisting of an Elasticsearch database cluster, a Kibana (47) server and a Filebeat (48) data collector.

**Figure 4:**
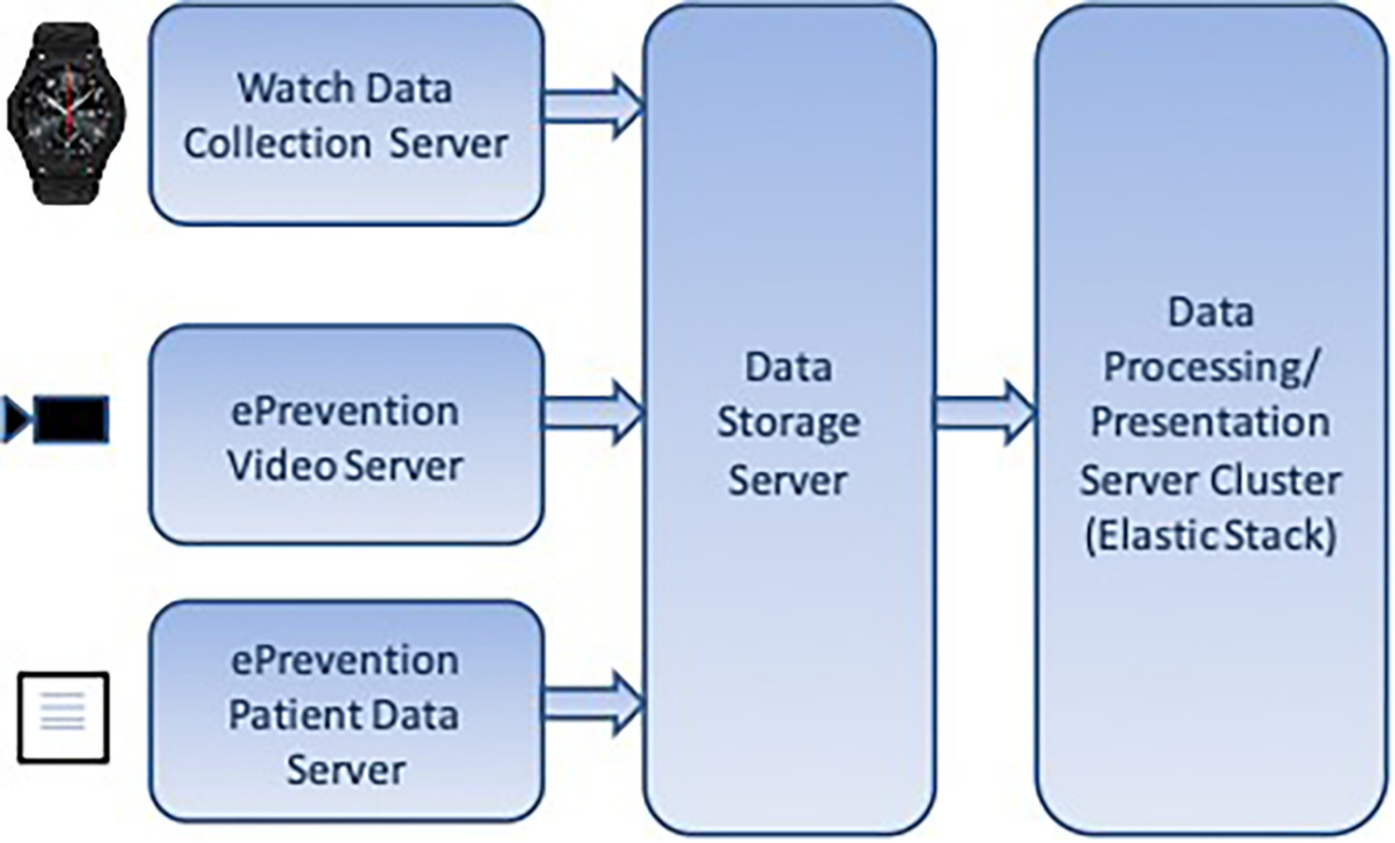
Data server diagram of the e-Prevention platform.

In the e-Prevention platform, the BioSignal Collection Server plays a central role in handling the biometric and motion data collected from wearable devices, such as smartwatches, and from tablets used during clinical sessions (Figure 4 **presents the diagram of Data Server).** These wearable devices generate a continuous stream of physiological signals like heart rate, RR intervals, sleep patterns, and activity levels, which need to be securely transmitted and processed in near real-time.

To achieve this, each smartwatch is equipped with a lightweight application that continuously gathers sensor data and transmits it securely over encrypted HTTPS connections. The application is designed to work autonomously and typically sends data when the watch is charging, in order to conserve battery life. On the tablet side, a dedicated mobile application has been developed to capture additional data, including video content and questionnaire responses. This application also uses secure TLS-encrypted communication to transmit data to the server infrastructure. Once received by the BioSignal Collection Server, each data packet is assigned a timestamp and a digital signature to ensure its authenticity and prevent any tampering or replay attacks. The server also performs data validation using predefined schemas to ensure only properly structured and expected data types are processed. This helps to defend against potential injection attacks or corrupt payloads.

To further secure the system, Role-Based Access Control (RBAC) is implemented at the API level, allowing only authorized users and services to access specific functions or data. Before being stored, the data is briefly cached in a secure buffer and then indexed in Elasticsearch. The Elasticsearch environment itself is hardened with encryption at rest, strict access policies, and is deployed in a private cloud network to limit exposure to external threats. Together, these mechanisms form a robust and secure pipeline for managing sensitive health information. They ensure that the data is not only protected from unauthorized access but also remains intact, verifiable, and available when needed. This approach directly addresses the challenges of continuous, real-time biometric monitoring, and builds on the cloud-based architecture outlined in the work of Maglogiannis et al. (3), which guided the development of our proposed system.

### 4.1 Selection of the cloud platform—security considerations in using elasticsearch

The e-Prevention system utilizes the Elastic Stack, particularly Elasticsearch, as the core engine for storing, aggregating, and querying biomedical and behavioural data collected from wearable devices and medical applications. Although Elasticsearch is widely known as a high-performance search and analytics engine, it is not a dedicated security framework. Therefore, its deployment in the e-Prevention architecture is supported by a comprehensive security strategy that ensures compliance with healthcare data protection standards and addresses the limitations of traditional database systems.

In this context, Elasticsearch contributes to the overall security framework through features such as real-time log analysis, system monitoring, and flexible access control policies. These capabilities are harnessed within a broader secure environment to meet the specific needs of a continuous monitoring platform. All communication to and from Elasticsearch is protected using HTTPS, ensuring secure transmission of sensitive data. The platform's built-in Role-Based Access Control (RBAC) is employed to enforce fine-grained access policies, ensuring that only authorized users can query or manipulate data. Furthermore, data integrity is preserved through the application of cryptographic hashing and digital signatures prior to storage. The system also implements audit logging, with logs visualized through Kibana, enabling real-time detection of anomalous behaviours or unauthorized access attempts.

In addition, the cloud infrastructure hosting the Elasticsearch cluster is configured to provide encryption at rest and high availability. Virtual machines managed by a secure cloud provider (GRNET) isolate the Elasticsearch services in a private network, inaccessible from the public internet except via authenticated administrative endpoints. These layered measures ensure that while Elasticsearch plays a central role in handling and indexing sensitive health data, the actual security of the e-Prevention system is achieved through the thoughtful integration of encryption, access control, integrity checks, and system observability—turning a general-purpose data engine into a secure foundation for continuous, privacy-sensitive patient monitoring.

### 4.2 User roles of e-prevention platform

The e-Prevention platform supports many user roles, for different purposes. Table 1—Role overview summarizes the available roles in the system.

**Table 1:** Role overview.

Role	Description
Patient	Collects BioSignal data from its smartwatch and uploads them to the platform.
	Records video interactions during doctor appointments
Doctor	Completes questionnaires with patients’ examination results.
	Observes patients’ historical data visualizations
Researcher (e-Prevention team)	Queries Elasticsearch to fetch patients’ data for analysis with machine or deep learning models.
	Creates custom visualizations of aggregated patients’ data
Administrator	Manages users’ access rights to e-Prevention resources.
	Manages registered smartwatch devices.
	Manages the e-Prevention platform’s collected data.
	Manages infrastructure components and performs maintenance activities.
	Monitors infrastructure for performance and security issues

### 4.3 Data flow

**Figure 5:**
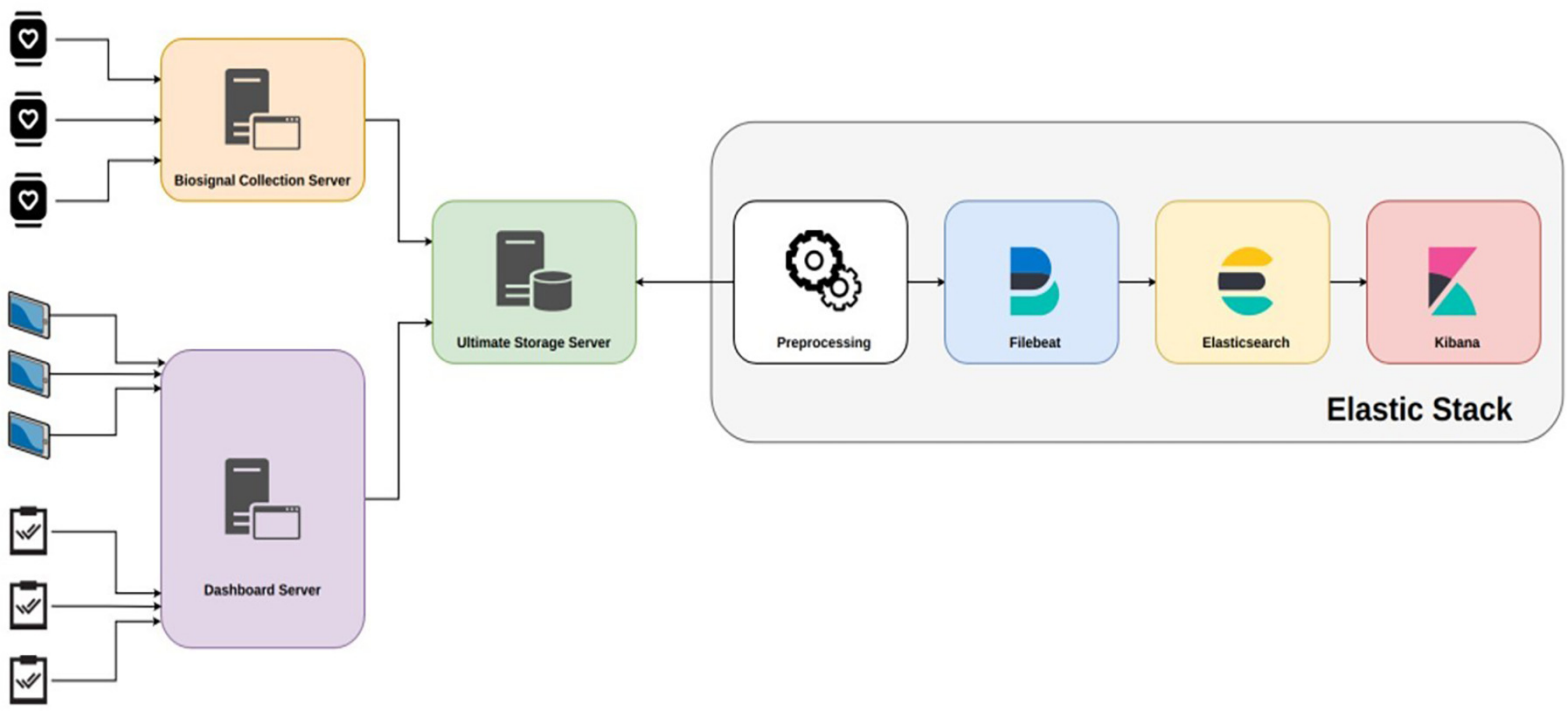
Data flow in e-Prevention system.

This section gives an overview of the data flow (from smartwatch, tablet, and questionnaire inputs to storage and processing in the e-Prevention system) Figure 5. Additionally, the dependencies between the sensor data and the planned modules for the application processor are presented.

The data flow can be separated into the high-level data flow between the patients, the BSN, the applications and the Platform, and the data flow of the application processor that must solve dependencies between sensor data and the internal modules that depend on the latest processed data of other modules. As it is obvious, the overall data flow is divided into three main stages:

1. Data collection: At this stage there are three data streams from various sources:
  • The data from smartwatches end up in the BioSignal Collection Server which forwards them to the Ultimate Storage Server for long-term storage.
  • The data from the video recordings end up in the Dashboard Server, which forwards them to the Ultimate Storage Server for long-term storage.
  • The data from the questionnaires are entered manually by the doctors in the Dashboard Server. On a weekly basis the application is forwarding them to the Ultimate Storage Server for long-term storage
2. Long-term data storage: At this stage, the data from all three sources have been gathered into the Ultimate Storage Server but are still in unified and raw compressed format.
3. Data processing, analysis, and visualization: At this stage, the data is processed, transformed into an appropriate format, and homogenized, serialized in time and visualized. This step includes the following sub-stages:
  • Preprocessing: Depending on the data source, they are transformed into a readable format by Filebeat.
    a) In the case of smartwatch biosensor data, they are enriched with smartwatch system metadata such as device ID.
    b) In the case of video recordings, metadata is generated that contains information such as the storage location of each video, the patient ID, etc.
    c) In the case of questionnaires, the information is decomposed per patient questionnaire, a format suitable for processing by Filebeat.
  • Filebeat: The Filebeat daemon is configured to locate data files of all categories by name and structure. It then performs various transformations on them and forwards them to Elasticsearch with an automatic mechanism.
  • Elasticsearch: Elasticsearch receives data from the Filebeat daemon and, after serializing it over time, stores it in its directory.
  • Kibana: Kibana performs user-defined queries on Elasticsearch data and draws real- time graphs with the results.

### 4.4 Summary and classification of general features and requirements of the e-prevention system

E-Prevention is a system for continuous patient monitoring, aiming to early diagnosis and detection of worsening events and treatment of patients suffering from psychotic disorders. The system assists this category of patients in their everyday life, and supports them in their everyday living, by monitoring them through a wearable BSN. The system features additional functionalities to support the specified security and privacy aspects. These features entail a set of functional and non-functional requirements. The functional requirements are the requirements of the system components and technology (hardware and software/application web-based apps) including the system features. The non-functional requirements are data protection and privacy. The requirements for the e-Prevention systems are classified in four categories (Whole systems, Sensors, Data Collection and Applications). Some of them are presented in the following Table (Table 2**—General Requirements of the e-Prevention system**).

**Table 2:** General requirements of the e-prevention system.

Requirement	Description	Category
The system will be prepared for continuous use.	System must be prepared to be used on a 24 × 7 basis, during extended time periods.	Entire system
System will be adapted to end users	The system must be designed for patients with psychotic disorders, but usable by any patient.	Entire system
The system will provide feedback to the end-users	The BSN and apps will include the transmission of information to the users.	Entire system
	Data collection and processing must provide complete information for each use case	
The system components will communicate wirelessly	System components will communicate within a wired BSN with some external components connected wirelessly (sensors)	Entire system
System must be fully developed in the users’ language	Initially: English	Entire system
System use will be easy to use and understand	All the system’s components that interact with the user must be user friendly and easy to understand	Entire system
System will be easy to learn	The cost of leaning the system features and functioning should be minimum. Short-term memory decline with age should be considered both at the app design and at the training phase.	Entire system
BSN will have a minimum autonomy of 24 h	BSN should have enough autonomy to operate for 24 h	Sensors
Sensor’s actuators will provide feedback to the user	Feedback will be done via smartwatch.	Sensors
	Feedback from sensors can be acoustic, haptic, or visual.	
BSN will include an integration of specific developed sensors and existing solutions	BSN will integrate existing wearables (e.g., smartwatch)	Sensors
The system will include physiological sensors	Physiological sensors will include heart rate and RR Intervals. It will collect information about dynamic health indicators	Sensors
The system will include environmental sensors	For example: noise, indoor air quality, distance	Sensors
System will collect data about environmental aspects	It includes noise, temperature, humidity	Data Collection
System will collect data about BioSignal measures		Data Collection
System will collect data from the video recordings		Data Collection
System will collect data from the questionnaires filled by the doctors		Data Collection
Doctors’ app will provide alerts	Information for doctors will be adapted	Application
Doctors’ app will include specific patient’s indicators and parameters	Parameters will be related to health status	Application
Doctors’ app will provide long-term detection	Information for doctors will be adapted from the assessment of the system	Application
Apps will provide the same means of use for all users	Consistency—Apps for patients, doctors, administrators etc. should have the same structure and organization	Application
App design will be appealing to all users	During the design process, a common style and specific variations depending on users’ needs, must be considered	Application
App interface will be simple	Complex environment should be avoided. The system should not require many user interactions.	Application
Apps will be easy to install and configure	The apps will be easy to install. Apps will allow configuration by the users. Configuration will be easy.	Application
Doctors’ app will be adaptable to the feedback of specific users		Application

## 5 Proposed security framework

### 5.1 Overview of security framework methodology

**Figure 6:**
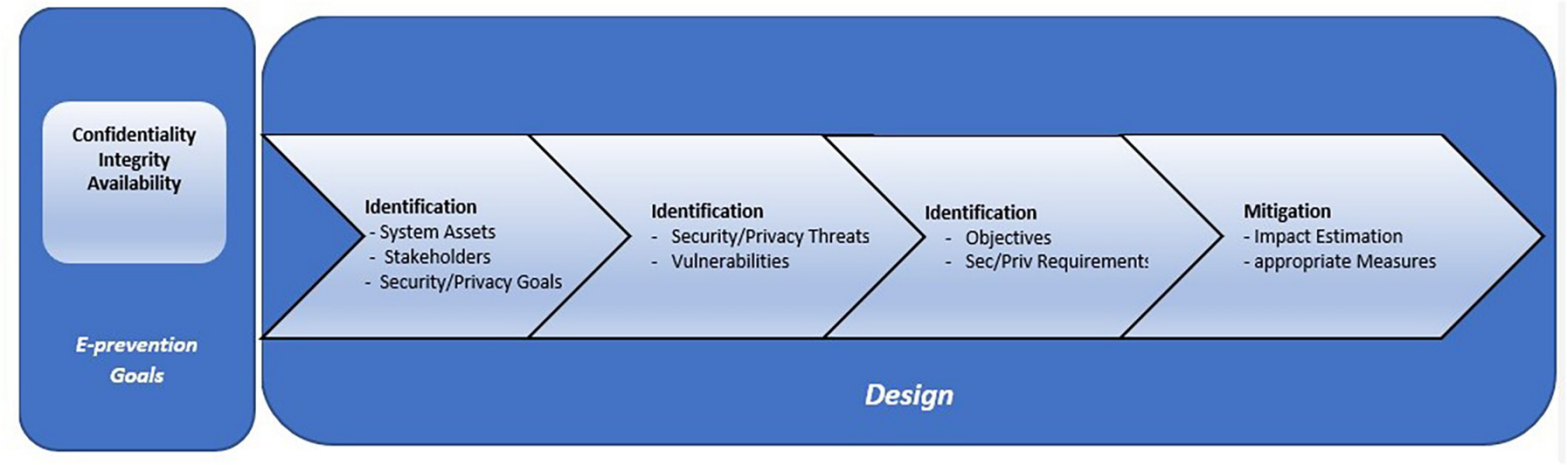
Proposed secure methodology.

As in fact, early identification of worsening symptoms in the early stages of the psychotic process, and early prevention of relapses have been found to contribute significantly to better outcomes of the disorder (49–51) and in preventing the catastrophic effects that relapses often have on patients' lives (52). Given that psychosis is evolving continuously, and relapse is a biological process that develops over time, it would be reasonable to anticipate variations in the behaviour of such biomarkers that are related to and probably precede the onset and/or worsening of such mental disorders. So, on the above basis, it would be possible to create an intelligent system that could continuously and passively measure human behaviour to detect these changes and predict psychotic relapses before symptoms are fully expressed. The main scope of the e-Prevention project is the development of an advanced application for monitoring and relapse prevention of patients with psychotic disorders. This is done by using long-term recording and analysis of biometric indicators with the active participation of patients themselves. In this section we present the design of our Secure Proposed Framework for Cloud services, which is based, in our previous publications on the security and privacy issues of Cloud-based health systems (53–58) and in our previous research on Healthcare applications (59–62). The proposed secure framework is applicable to the early stages of the e-Prevention system design. The whole list of security and privacy requirements to be fulfilled from the e-Prevention applications, are derived from the proposed Security Framework. So, on the above basis, it would be possible to create a secure, intelligent system that could continuously and passively measure human behaviour to detect behavioural changes and predict psychotic relapses before symptoms are fully expressed. In the following sections, we will describe the steps of the proposed Methodology, that we should follow during the Design process of the e-Prevention system to cover security and privacy requirements. The proposed Methodology comprises of the following four stages, depicted in Figure 6.

**Step 1: Identification of System Assets, Stakeholders and Security—Privacy Goals of the e-Prevention system**: The purpose of this step is the definition of the boundaries of the under-study e-Prevention system. In other words, at this step, the data that will be defined are the important affected assets of the e-Prevention system, the classification of stakeholders, the communication channels and relations between components, the security issues that need to be addressed, and furthermore, the specification of high-level security and privacy goals of the e-Prevention stakeholders. Unique identification of information assets is the first task to ensure the protection of information. As an asset is basically any entity of value, the assets that are critical for the e-Prevention system and operations will be clarified. It has been decided by the e-Prevention team and the users that the only purpose of processing that will be considered during the security and privacy analysis is PP1: Evaluate medical data to improve patients' health. Thus, all the analysis of the e-Prevention system will be based on this purpose of processing. In addition to the identified assets, the second task of this step will be the identification of the communications channels involved in the e-prevention architecture (e.g., USB, Bluetooth, Wi-Fi etc.) which are potential risks sources and thus should be also protected. Added to the identification of communication links, the third task will be the identification of the users' categories, in other words, the types of stakeholders of the e-Prevention System and the classification of them. In the e-Prevention system, the identified user categories (or the types of stakeholders) are classified in the following four roles: patients, administrators, doctors, and e-Prevention team. Finally, the last task of this Step is, the identification of the security and privacy goals that the e-Prevention system should meet. Based on the functional requirements derived for the e-prevention system, the goals, and functionalities that the system should fulfil and the stakeholder's needs, the security and privacy goals that the e-Prevention system should meet are the following: (1) confidentiality of stored and transmitted data, (2) integrity of the stored and transmitted data and (3) availability of the e-Prevention services.

**Step 2: Identification of Potential Security and Privacy Threats and existing related Vulnerabilities of the e-Prevention system:** During this Step, a definition of the potential threats and the related vulnerabilities that are going to be specific for the e-Prevention infrastructure components will be performed. These defined threats and existing vulnerabilities will be used from a devoted risk analysis that will be used at this step. A risk analysis or risk assessment identifies and characterizes the elements that compose the risk (analyze the threats with the corresponding assets, vulnerabilities, impact, existing security measures). The main aim is to ensure that necessary security and privacy objectives are integrated into the design part of architecture. Thus, risk analysis is usually seen as extremely important in analyzing the security features needed by a system. *Α* vulnerability is defined as a weakness which can be exploited by a threat actor, such as an attacker, to perform unauthorized actions within a computer system and a threat is a potential negative action facilitated by a vulnerability that results in an unwanted impact to a computer system or application. More analytically, the operations that will be performed at this step are a list with the relevant attack methods against security and privacy, the characterization of the threats, the identification of the security and privacy vulnerabilities of the entities, an estimation of vulnerability level, the identification of the potential security and privacy threats and the prioritization of these threats according to the probability of their occurrence. A threat is an event or a circumstance which could cause a security incident. This task involves assessing threats and security risks associated with the e-Prevention system. Through a threat model, the major threats in the e-Prevention system as it is a WBAN system were presented and identified by the e-Prevention team, by e-Prevention end users, and by other health researchers' previous experience/publications.

**Step 3: Security and Privacy Requirements Analysis of the e-Prevention system:** At this step, based on the detection of vulnerabilities, an identification of the security and privacy objectives will be presented. Thus, if the vulnerabilities are reduced, then the potential risk on the identified entities will be also reduced. The next task of this step will be the definition of the security and privacy requirements that should be satisfied for the e-Prevention purpose of processing: PP1: Evaluate medical data to improve patients' health. These requirements include the most important aspects of e-Prevention's security. For the identification of the appropriate requirements, the following process will be followed: based on the previously defined security and privacy goals (Confidentiality, Integrity, and Availability), the respective threats and vulnerabilities were identified. Then, as the specific threat could exploit the specific vulnerability, for each threat and vulnerability the specific affected e-Prevention assets will be examined. This identification will guide us to the specification of the security and privacy properties that should be protected and, thus, to the elicitation of the appropriate requirements of the e-Prevention project. The above process ensures the mitigation of the identified threats and thus the coverage of the appropriate protection level for the e-Prevention system. In the following *Τ*able 3, the derived security and privacy requirements for the e-Prevention system are listed.

The specific list of these 15 requirements along with the list of general requirements identified and presented previously derived through the application of this Framework. The satisfaction of the requirements will ensure that e-prevention system will exhibit all the appropriate countermeasures against potential security threats.

**Step 4: Identify Security and Privacy Measures—Mitigation:** The aim of this step is the identification and prioritization of the appropriate technical security and privacy measures that fulfill the requirements (needs) of the e-Prevention system presented in the previous Table (Table 3). These measures should be implemented to reduce the identified risks to acceptable levels. Thus, during this stage, all the requirements elicited in previous steps will be evaluated to eliminate risks and any conflicts prior to the selection of security and privacy measures. Therefore, the main output of this step is the process of selecting and implementing measures to modify risk. The idea behind this is to establish guidelines and recommendations for Operators, Manufacturers and Users, to properly respond to potential cyber-attacks and ensure the overall security of the e-Prevention environment. This step consists of the development of some strategy to address each of the risks analyzed previously. In other words, it establishes a series of actions to reduce, retain, avoid, or transfer data risk according to a defined treatment plan, it proposes solutions to security problems.

**Table 3:** Security and privacy requirements for the e-prevention system.

Requirement ID	Requirement
RE1	Data Confidentiality during transmission
RE2	Data Integrity during transmission
RE3	Availability of the required resources
RE4	Data Authentication
RE5	Interoperability of e-Prevention software components
RE6	Secure deployment of e-Prevention software
RE7	Authorization: only authorized users can access the e-Prevention system
RE8	Access control
RE9	Non-repudiation
RE10	Data Freshness
RE11	Accountability
RE12	Anonymity
RE13	Key management
RE14	Recovery of crucial e-Prevention assets after natural disasters and other physical threats
RE15	Reliability and security of the e-Prevention software development process

### 5.2 Enforcement of confidentiality, integrity, and availability (CIA)

While the e-Prevention framework references the principles of confidentiality, integrity, and availability (CIA), this section provides a detailed overview of how these are technically enforced through integrated mechanisms within the system architecture.

The confidentiality is achieved by encrypting all communication channels among wearable devices, cloud servers, and Elasticsearch engine through HTTPS encryption with automatic seamless HTTP to HTTPS redirection not to have insecure transmission. In addition, all stored data is encrypted either through native disk-level encryption provided by the GRNET cloud infrastructure or through Elasticsearch-compatible encryption plugins where appropriate. Role-based access control (RBAC) is implemented to secure access to sensitive endpoints so that only authorized users with the appropriate roles and components can access or modify covered resources.

Authentication is utilized in a multi-layered form. Smartwatches authenticate every session of data transfer using specific API tokens provided by the BioSignal Collection Server. Username/password credentials are utilized for dashboard logon and video data interactions with robust password policy and optional multi-factor authentication (MFA) enforced. Session integrity is secured through preventing simultaneous logons and enforced credential rotation. These authentication controls prevent unauthorized access and allow session accountability.

For data integrity to be ensured, all sensitive input data undergoes digital signing and cryptographic hashing before storage. They make sure that tampering or alteration—both in transit and at rest—can be identified. Component-to-component communication within the system is also secured with TLS certificates provided by Let's Encrypt to guarantee message origin authenticity as well as content integrity. In addition, user activity, data transmission and processing audit logs are searched in Elasticsearch and monitored and stored to identify real-time anomalous behaviour using Kibana dashboards.

Availability is ensured through the operation of the system on high-availability cloud virtual machines in the GRNET infrastructure. Redundant servers, load balancers, and failover ensure services are available even in the event of hardware failure or cyber-attack. Cold storage backups and the segregation of mission-critical services into logically distinct entities improve resilience and system availability.

Collectively, these measures place the CIA triad on every dimension of the e-Prevention platform to provide a scalable and secure foundation for security critical to supporting real-time continuous monitoring of mental health data.

## 6 Implemented features in the e-prevention system

In Table 4 the security and privacy requirements satisfied or not at the e-Prevention system after the application of the proposed secure framework are presented. Moreover, an explanation of the implemented measures for the satisfied requirements is described. The satisfaction of the above requirements will ensure that the e-Prevention system will protect the security of the patients and will exhibit all the appropriate measures against security threats.

**Table 4:** Satisfaction of the requirements after the application of the secure framework.

Requirement ID	Requirement	Satisfied/Not Satisfied
RE1	Data Confidentiality during transmission	Satisfied
RE2	Data Integrity during transmission	Satisfied
RE3	Availability of the required resources	Satisfied
RE4	Data Authentication	Satisfied
RE5	Interoperability of e-Prevention software components	Satisfied
RE6	Secure deployment of e-Prevention software	Satisfied
RE7	Authorization: only authorized users can access the e-Prevention system	Satisfied
RE8	Access control	Satisfied
RE9	Non-repudiation	Satisfied
RE10	Data Freshness	Satisfied
RE11	Accountability	Satisfied
RE12	Anonymity	Satisfied
RE13	Key management	Satisfied
RE14	Recovery of crucial e-Prevention assets after natural disasters and other physical threats	Satisfied
RE15	Reliability and security of the e-Prevention software development process	Satisfied

The appropriate measures that fulfil the requirements of the e-Prevention system presented in the previous Table (Table 4) are the following:

• RE1—Data confidentiality: Denotes the protection of confidential data from exposure, which is considered a crucial issue in a WBAN. To satisfy this requirement, e- Prevention is provided on a secure communication channel between secured WBAN nodes and their coordinators with encryption. For this purpose, all the Cloud components are properly configured to use the HTTPS protocol for all connections. Additionally, if a connection attempts to use the HTTP protocol, then the communication is automatically redirected to HTTPS. Moreover, the e-prevention system uses strong authorization, deploys appropriate access control mechanisms and only authorized users' access to data.
• RE2—Data Integrity: Data integrity is mainly concerned with how reliable, accurate and ultimately valid the data of an application is. Current e-prevention smartphone platform supports device and/or application integrity checking features that should be leveraged to check the integrity of the device and application. A threat that relates to data integrity is the threat of Tampering that refers to malicious modification of data or processes and may occur on data in transit (e.g., from Body Area Networks to Local Area Networks), on data at rest, or on processes. To satisfy the requirement of Data Integrity, the e-Prevention system uses certificates through which encrypted communication takes place and have been issued by Let's Encrypt. Furthermore, communication links between system components are ensured by using protocols that provide message integrity and confidentiality. Lastly, the e-prevention system uses data hashing and signing. All confidential data be hashed and signed to ensure that the data is valid (untampered and came from the correct/expected source) and strong authorization with appropriate access control mechanisms such as role-based access control (RBAC) with least privileges and separation of duties principles.
• RE3—Availability: Since e-Prevention system carries important and highly sensitive data, it is required that such network be available at any time for the patient use. For this reason, it is important to switch the operations to another server in case of an availability loss incident. To satisfy this requirement, the server side of e-Prevention has been deployed in virtual machines, running in the Cloud offering of GRNET. By doing this, GRNET as a Cloud provider can guarantee that the virtual machines and the networking will be available and operational at any time. In addition to this, to satisfy the requirement of availability, the e-prevention system uses strong authentication- the user is authenticated to the system using a strong password policy, biometrics and multi-factor authentication mechanisms. Furthermore, the e-prevention system uses the method of encryption. All credentials are encrypted, and it is ensured that credentials do not traverse the wire in clear text form. Lasty the e-prevention system uses cryptographic protocols such as TLS/SSL to ensure secure (encrypted) communication between system components.
• RE4—Data Authentication: One of the main requirements for all medical applications is data authentication, since they must verify that the source of received data is a known trust center rather than a malicious actor. Identification of the users is critical as authentication is the foundation of access control as is audit logging. Continuous authentication is required to repeatedly verify that the device holder is the person initially authenticated. Patient smartwatches are connected to e-Prevention by
• Unique API tokens which are issued by the BioSignal Collection Server. Their management and registration are also done by the administrator through this component. Tablet devices are connected to the Dashboard Server with the usage of username/password credentials distributed to the patients during their registration to the platform.
• RE5—Interoperability: The e-Prevention platform is a multicomponent system. To guarantee that interoperability is respected, each component's role has been strictly defined and documented. To be more specific, all data flows and actions have been identified and assigned to specific components. To ensure that interoperability is achieved in a secure way, a private network has been created for internal communication between the e-Prevention components. This network is not accessible on the Internet, and it can be accessed only by the administrator through a jump host.
• RE6—Secure Software: To ensure that the deployment of the e-Prevention software is secure, it is installed by using the credentials provided by the Cloud operator. In addition, a big part of the e-Prevention infrastructure has been configured as code and can be easily redeployed from zero. Additionally, the e-Prevention platform relies on open-source software for its various components to guarantee that all the best practices have been followed during the development lifecycle of the software in use.
• RE7—Authorization Schema: To exhibit a fine-grained authorization schema for connected users to e-Prevention, an RBAC mechanism has been put in place. Through this mechanism, the users, after creating a unique account and password, fall into categories. Depending on the category to which it belongs, each user can perform different actions in the system. These categories are the translation of the e-Prevention roles which have been described in the previous section.
• RE8—Access Control: Access control refers to physical access to the location of the infrastructure where software is running. This requirement is covered by running the e-Prevention platform in the Cloud, since access to the physical servers in data centers, where e-Prevention virtual machines are running, is monitored, and guarded by the Cloud provider. On the other hand, remote access to the virtual e-Prevention infrastructure is allowed *via* secure shell protocol (SSH). Extensive care has been taken in the infrastructure so that: a) access *via* SSH is allowed only with the use of the private key of the administrator and no one else's, b) access is done through two steps: The administrator must first log in to a jump host and then connect through it to the rest of the virtual infrastructure.
• RE9—Nonrepudiation: Non-repudiations is the assurance that the sender of information is provided with proof of delivery, and the recipient is provided with proof of the sender's identity, so neither can later deny having processed the information. The e-Prevention system fulfils this requirement with the use of secure audit trails. All activities such as successful and unsuccessful authentication and sensitive data (e.g., cookies and authentication credentials) are logged and recorded in the e-prevention system.
• RE10—Data Freshness: Data freshness refers to how up to date the data in a report is. The e-Prevention platform is a live capturing system that collects data from patients on a 24-hour basis. To be able to withstand smartwatch synchronization issues and asynchronous video and questionnaire uploads by patients and doctors, the processing phase uses a different cycle. To be more specific, the processing of the collected data happens periodically in a 3-day sliding window. This delay is essential for the correct operation of the platform while it does not affect the freshness of the data.
• RE11—Accountability: Accountability means making sure every action can be tracked back to a single person, not just a group or ID. And it requires more culture change and needs to be handled with a light touch. To implement accountability, every account is unique and belongs to a single person or component of e-Prevention. In addition, simultaneous active sessions with the use of the same account are prohibited by design. Finally, no default passwords are used, and e-Prevention administrator is the only person who holds the admin credentials of the e-Prevention components, which are stored in a secure password vault.
• RE12—Anonymity: To ensure that the data in the e-Prevention system are anonymized and cannot be linked to a physical person, each patient has been assigned a random personal identifier with which its data are tagged. This identifier is the only unique characteristic of each patient and only doctors are aware of the correlation of their patients. The e-Prevention platform can run Personal Identifiers such as name, age, weight etc. and through a data anonymization process retain the data but keep the source anonymous. In addition to this, e-Prevention contains data from fake patients to enhance anonymity even further.
• RE13—Key Management: To fulfill this requirement the e-prevention system uses only the keys for the purpose they were intended. More analytically, it restricts the permissions on each key so that it may only be used for a specific purpose (e.g., encryption, decryption, verification, etc.). In case that the key must be distributed to another system, it uses various “key block” formats that bind the permissions to the key. Furthermore, in the e-prevention system all key management operations are performed according to strict and well-defined processes. These processes are in place to manage the consequences of a key that is suspected or known to be compromised. Lastly, the e-prevention system uses a centralized key management system to keep data with secure and efficient key generation, storage, and distribution.
• RE14—Recovery: The term recovery refers to the ability to resume operation after natural disasters and other physical threats. By running e-Prevention in the cloud,
• Recoverability is guaranteed, since any hardware failure or emergency is handled by the cloud provider who can migrate virtual machines between physical servers, data centers or regions, without interrupting their operation. To further boost the resilience of e-Prevention, the cloud components are replicated and access to them is managed by network Load Balancers. Another measure to boost recoverability is the cold storage of data in Ultimate Storage Server which is also physically located in a separate data center than the other components of e-Prevention.
• RE15—Reliability and Security: Reliability is defined as the probability that a given system operates properly for a specified period. To enhance reliability of e-Prevention, its system logs are collected in its Elasticsearch component and are monitored through the Kibana visualization platform by the administrator. By actively monitoring system data, e-Prevention administrators can identify and classify potential malicious or abnormal activities in every cloud component of e-Prevention. To boost security, a policy of strong passwords has been applied as well as short expiration and request for password renewals. In addition, login attempts, and active user sessions are monitored and analyzed by e-Prevention administrator.

## 7 Discussion

### 7.1 Limitations and future work

Though the suggested framework reflects a broad cross-section of the security features fitting the CIA paradigm, the performance has not yet been statistically evaluated using empirical measures or simulated. Rather, the system aims for low-latency and real-time like response based on published performance profiles for Elasticsearch and the other Elastic Stack components.

Elasticsearch, for instance, has attained sub-second search latency in earlier installations by optimal indexing and shard plans in the right configurations. TLS cluster member communication provides end-to-end encryption for data transfer with usually less than 10 ms overhead per query within optimally provisioned cloud setups. Lastly, Filebeat's minimalist architecture and system ingestion pipeline optimization minimizes CPU and memory loads during uninterrupted data ingestion. Audit logging and role-based access control are used to offer responsiveness and security without introducing substantial delays in user interactions.

However, it is important to note that latency, throughput, and fault tolerance under load have not been measured or simulated in this work. These are the premise of follow-up studies, which will involve a thorough performance benchmarking of the platform for different data ingestion and query loads. Comparative studies with other existing competing e-health monitoring frameworks are also anticipated to further strengthen the practicability and scalability of the proposed design in actual clinical environments.

### 7.2 Ethical and legal considerations

Since the e-Prevention system entails ongoing monitoring of sensitive patient information, such as biometric data, behaviour patterns, and mental health status, ethical functionality and legality are foremost in its development. The platform has been developed with great priority placed on privacy, openness, and user control, and possesses inherent controls to become GDPR and HIPAA compliant in the future. In this instance, explicit informed consent must be sought from all the participants before data collection. This involves clear communication of what kind of data will be collected, how it will be utilized, and who will be acquainted to it. Patients are also free to withdraw their permission or ask for erasure of data at any time, which falls in line with the concept of data ownership and enhances ethical utilization of health data. Though complete regulatory compliance remains in the works, the architecture itself already enforces best practices under GDPR, including data minimization, role-based access control, and encryption of data in transit and at rest. Personally identifiable information (PII) is isolated from clinical and biometric data through pseudonymization, and sensitive information access is strictly limited to approved healthcare providers.

Among the essential building blocks of the project's future growth is to do a thorough data protection impact assessment (DPIA) and institute policies and measures required in order to obtain complete GDPR conformity. Likewise, further procedural controls will be enforced for HIPAA compatibility should the system be planned to be rolled out to U.S.-based deployments. With forward-looking inclusion of ethical considerations and enforcement of privacy-oriented design patterns, the e-Prevention platform builds the groundwork for a secure, user-sensitive, and regulation-friendly solution for mental health monitoring.

### 7.3 System constraints and scalability challenges

As a real-time monitoring platform for continuous biometric and behavioural, the e-Prevention system is subject to various architectural and operational limitations which should be addressed in future revisions. Computational overhead, especially during times of heavy data ingestion and processing, is one key area of concern. Although modules such as Filebeat and Elasticsearch are designed for high-throughput scenarios, constant streaming from numerous patients for long periods of time can result in higher CPU and memory consumption, particularly with real-time alerting and analytics turned on. Scalability in terms of storage is also something to be worried about, with prolonged monitoring leading to extensive storage of high-resolution time-series, video recording, and inputs from clinicians. It is currently based on cloud-based virtual machines and cold storage, but retention policies, compression, or hierarchical storage management (HSM) may be required in the future to balance performance and cost. Moreover, user scalability, query scalability, and concurrent access are also issues with growing users, queries, and concurrent access among researchers, clinicians, and patients. Providing low-latency responsiveness and system responsiveness under load necessitates strategic backend resource coordination, sharding strategy within Elasticsearch, and potentially horizontal scaling of services.

Although the design is keeping modularity and cloud hosting in view, there will be a requirement for continuous stress testing, load balancing, and auto-performance tuning to ensure fault tolerance of the system in actual clinical deployment scenarios.

## 8 Conclusions and next steps

Since Wireless Body Area Network (WBAN) systems are being used more and more in the collection of sensitive patient information, they need to have a secure and resilient architecture to ensure the integrity and confidentiality of the communication they create. Health care services enjoy nearly real-time surveillance and transmit sensitive medical data of patients who are extremely worried about data confidentiality and privacy. But the ubiquitous and distributed nature of WBANs leaves them vulnerable to a broad attack surface, and security and privacy become necessary but daunting. objectives.

The e-Prevention platform, being an instance of a WBAN-based remote patient management system for people afflicted with mental illnesses, entails continuous sharing and reaping of high volumes of biometric and behavioural information. The aim of this paper was to introduce a secure cloud repository architecture on top of Elasticsearch, integrated at the initial phase of system design, to fulfil security and privacy needs in a complete sense. The architecture not only provides secure data handling and excellent precision in real-time health monitoring but also tries to fulfil functional as well as non-functional requirements dictated by healthcare professionals and end users.

The system is secure with confidentiality via encrypted communication and rules of access, integrity via the use of cryptographic hashing and digital signatures, and availability via the use of strong authentication like RBAC, biometric authentication, and multi-factor authentication. The data is encrypted in transit at every level from the medical device to the cloud storage unit and only made accessible to approved personnel.

To envision the required framework, we referred to existing WBAN security solutions, identified key deficiencies in mental health monitoring systems, and debated limitations of current cloud architectures with an eye towards this purpose. Due to a lack of any current framework for mental health monitoring systems, we have devised a set of guidelines to steer future work toward developing a GDPR-compliant solution to deploying secure, privacy-enforcing cloud services using Elasticsearch.

Near-term future work will involve formal verification of the framework outlined above, including performance benchmarking (latency, throughput, and fault tolerance), threat modelling, and live stress testing. Moreover, we intend to utilize a Technology Readiness Level (TRL) roadmap to organize this development. This will enable system maturation from conceptual framework to verify, deployable architecture for secure and scalable mental health monitoring in real-world settings.

## Data Availability

The original contributions presented in the study are included in the article/Supplementary Material, further inquiries can be directed to the corresponding author.
